# Polymer Gels Exhibiting High Pressure-Sensitive Adhesion to Polytetrafluoroethylene

**DOI:** 10.3390/polym18040538

**Published:** 2026-02-22

**Authors:** Toshiya Yamasaki, Yuchen Mao, Hiroshi Ito, Jin Gong

**Affiliations:** 1 Department of Organic Materials Science, Graduate School of Organic Materials Science, Yamagata University, 4-3-16 Jonan, Yonezawa 992-8510, Yamagata, Japan; t241026m@st.yamagata-u.ac.jp; 2 Dishui Lake Laboratory, Shanghai Dianji University, Lingang Campus, 300 Shuihua Road, Pudong New Area District, Shanghai 201306, China

**Keywords:** adhesion, pressure-sensitive, polymer gel, polytetrafluoroethylene (PTFE), adhesion mechanism

## Abstract

Polytetrafluoroethylene (PTFE) is attractive for high-frequency communications but adheres very poorly to other materials due to its very low surface energy. Conventionally, surface treatments of PTFE are used to increase the polarity of the PTFE surface and enable bonding to materials with increased surface free energy. However, surface treatments are difficult to scale, can damage surfaces, and often lack reproducibility. Therefore, developing a material that can make PTFE adhere well to other materials without surface treatment is highly desirable. In this study, we aimed to develop a new material with strong adhesion to PTFE. We synthesized three polymer gels from dodecyl acrylate (DA) and 2-(dimethylamino) ethyl acrylate (DMAE): the homopolymer gels PDEAE and PDA, and the copolymer gel P(DEAE-*co*-DA). The copolymer gel P(DEAE-*co*-DA) exhibited high pressure-sensitive adhesion to PTFE, recording the highest adhesive strength (*F* = 430.0 N/m) and the highest peel energy (*G* = 713.4 J/m^2^) compared to the homopolymer gels PDEAE and PDA. Mechanical testing showed PDEAE had the greatest strength and toughness, PDA balanced stiffness and extensibility, and P(DEAE-*co*-DA) was the most flexible and extensible. The P(DEAE-*co*-DA) with the smoothest surface (Sz ≈ 0.176 µm) showed the highest *F* and *G*, implying that surface roughness did not contribute significantly to the interfacial adhesion between the gels and the PTFE. Based on the surface free energy $\sigma_{s}$ and work of adhesion $W_{a}$ values, the adhesive strength to PTFE was predicted to be PDEAE > P(DEAE-*co*-DA) > PDA, but the measured *G* in peel tests contradicted this, indicating that the gels’ viscoelastic deformation and energy dissipation dominate the measured *F* and *G*. The frequency-dependent viscoelastic data and relaxation times *τ* and activation energies $E_{a}$ suggested optimal adhesion requires a balance of adhesion (mobility for energy dissipation (short *τ*, low $E_{a}$)) and sufficient cohesion (high G′). P(DEAE-*co*-DA) achieved this balance, explaining its high measured *F* and *G*. With precise control of polymer chain mobility, the adhesion of P(DEAE-*co*-DA) gels can likely be improved further. Future work will employ block copolymerization and monomer-ratio control to tune molecular motion and enhance adhesion to PTFE.

## 1. Introduction

Pressure-sensitive adhesives (PSAs) exhibit sufficient adhesive performance for practical use when they bond instantly to a substrate under light pressure [1], and they can usually be easily removed from the substrate [2,3]. Because of this convenience, PSAs are used in a wide range of fields, including the automotive and aerospace industries, information and telecommunications industry, and biology [4,5,6,7]. Of these, the information and telecommunications industry has grown especially rapidly in recent years. Faster signal transmission speeds have followed this growth, and in order to support very-high- speed transmission, operation at high frequencies has become increasingly important [8]. In high-frequency communication technology, the electrical properties of insulating materials strongly affect signal transmission efficiency and loss, which in turn determine the overall communication performance [9,10,11,12]. Materials with lower dielectric constants produce less signal attenuation and distortion, and this property is particularly important for communications in the frequency range of several GHz and above. In this context, polytetrafluoroethylene (PTFE) has a low dielectric constant and a low dielectric loss tangent, delivering excellent transmission performance in the high-frequency band. PTFE also has very high heat resistance, little degradation due to temperature changes, and very high chemical stability, enabling stable long-term performance even in harsh environments. Therefore, PTFE is well-suited as a substrate material for high-frequency communication equipment [13,14].

However, because of the strong bonding of fluorine atoms, PTFE has quite low surface free energy and consequently extremely poor adhesion to other materials [15]. To solve this problem, a variety of surface treatments, such as chemical etching, corona discharge, plasma treatment, and flame treatment, have been used to increase the polarity of the PTFE surface and enable bonding to materials with increased surface free energy [16,17,18,19]. However, surface treatments are difficult to scale for manufacturing, can damage the surface, and often lack reproducibility because they may require the use of organic solvents. Therefore, it is highly desirable to find a material that can make PTFE adhere well to other materials without surface treatment, especially from an industrial application perspective.

Polymeric materials are a class of adhesives commonly used across a wide range of industrial fields because they possess both elastic and viscous properties, providing strong adhesion and good mechanical strength [20,21]. In this study, we aimed to develop a new material with high adhesion to PTFE. We synthesized three polymer gels from two monomers, dodecyl acrylate (DA) and 2-(dimethylamino) ethyl acrylate (DMAE): two homopolymer gels, poly(2-(diethylamino)ethyl methacrylate) (PDEAE) and poly(dodecyl acrylate) (PDA), synthesized from DEAE and DA, respectively; and one copolymer gel, poly(2-(diethylamino)ethyl methacrylate-*co*-dodecyl acrylate) (P(DEAE-*co*-DA)), copolymerized from DEAE and DA. We evaluated the adhesive properties, surface structure, mechanical properties, and viscoelastic properties of the three gels (PDEAE, P(DEAE-*co*-DA) and PDA), and investigated their adhesion mechanisms to PTFE.

## 2. Materials and Methods

### 2.1. Chemicals and Reagents

The monomer DEAE and the initiator 2-hydroxy-2-methylpropiophenone (HMPP) were purchased from Tokyo Chemical Industry Co., Ltd., Tokyo, Japan. Another monomer DA and crosslinker 1,6-Hexanediol dimethacrylate (HDDA) were provided by Kanto Chemical Co., Inc., Tokyo, Japan. All of the above agents were used without further purification. The PTFE plate (thickness: 2 mm) and PTFE film (thickness: 100 μm) were purchased from MonotaRO Co., Ltd., Osaka, Japan.

### 2.2. Synthesis of Adhesive Gels

**Scheme 1:**
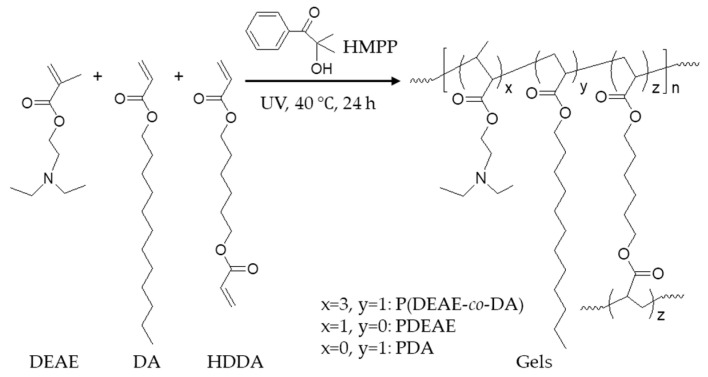
The synthesis scheme of three gels discussed in this work: a copolymer gel of P(DEAE-*co*-DA), and two homopolymer gels of PDEAE and PDA.

**Figure 1:**
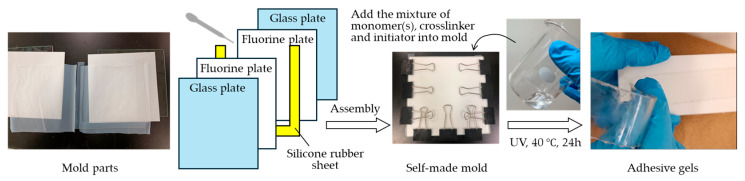
The synthesis process of gels shown in Scheme 1.

A copolymer gel of P(DEAE-*co*-DA) was synthesized using DEAE and DA as monomers, HMPP as the crosslinker and HDDA as the initiator. The molar amounts of DEAE, DA, HMPP and HDDA were fixed at 3.0 mol, 1.0 mol, 0.002 mol and 0.004 mol, respectively. For comparison, two homopolymer gels of PDEAE and PDA were also synthesized from DEAE and DA, respectively, using the same crosslinker and initiator. All gels were synthesized via photo-radical bulk polymerization according to the following procedure. The monomers, crosslinker, and initiator were placed in a 50 mL snap-cap vial and stirred for 15 min. The resulting mixture was then poured into a self-made mold and irradiated under a UV lamp at 40 °C for 24 h. The overall synthesis process is shown in Scheme 1 and Figure 1.

### 2.3. Characterization

#### 2.3.1. 3D Measuring Laser Microscopy

The morphology and surface roughness of the three gel sheets: PDEAE, P(DEAE-*co*-DA) and PDA, as well as the PTFE plates were observed and analyzed using a 3D measuring laser microscope (LEXT OLS4000, OLYMPUS, Tokyo, Japan). Surface roughness parameters, including the arithmetic mean height (Sa), root mean square height (Sq), and maximum height (Sz), were measured and averaged over five different locations on each surface.

#### 2.3.2. Tensile Test

The mechanical properties of the PDEAE, P(DEAE-*co*-DA) and PDA gels were determined by tensile tests using a versatile tensile testing machine (AGX-X, SHIMADZU, Kyoto, Japan). The specimens were dumbbell-shaped and cut using a laser cutting machine, with dimensions of 35 mm × 6 mm × 1 mm. The specimens were elongated at a constant rate of 100 mm/min until the specimens broke at room temperature. The tensile strength at break and yield, elongation at break, Young’s modulus, and toughness were evaluated from the tensile stress–strain curves. Testing was run on a minimum of five specimens. The reported data were the average of the results of five specimens.

#### 2.3.3. Peel Test

**Figure 2:**
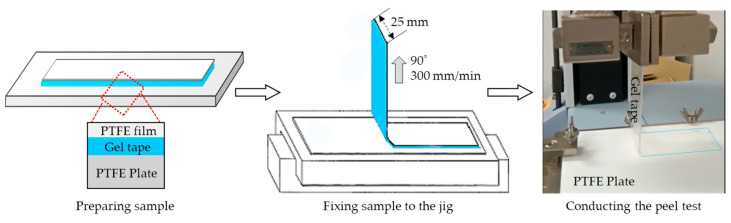
The schematic illustration of the 90° peel test. The gel tape is shown in blue. The sample is prepared by sandwiching the gel tape between a PTFE plate (thickness: 2 mm) and a PTFE film (thickness: 100 μm). The test is conducted using gel tape with a width of 25 mm at a peel speed of 300 mm/min.

The peel test is the primary measurement method that numerically captures adhesive strength. The pressure-sensitive adhesive strength of the PDEAE, P(DEAE-*co*-DA) and PDA gels was measured by a 90° peel test using a universal tensile testing machine (AGX-X, SHIMADZU, Kyoto, Japan). Test specimens were in the form of strips, cut out using a laser cutter to 300 mm × 25 mm × 1 mm. The gel tape specimens were pulled off at a constant rate of 300 mm/min at room temperature until the specimens were pulled off PTFE plates. A schematic illustration of the peel test is shown in Figure 2. The adhesive strength (*F*) was calculated from the measured force–displacement curve by averaging the peeling force per unit width over the displacement range of 25–75 mm. The peel energy (*G*) is obtained from the same force–displacement data by dividing the total peel energy (*E*) by the actual peeled area (*A*) (Equation (1)) [22,23].

(1) $$G=\frac{E}{A}=\frac{\int_{0}^{L}F\left(x\right)dx}{w\times L}$$

*E* is defined as the area under the force–displacement curve. *L* is the peeled length, and *w* is the specimen width. Five specimens were tested for each gel, and the reported values are representative results close to the mean of the five measurements.

#### 2.3.4. Contact Angle

Contact angle measurements were performed on PTFE, PDEAE, P(DEAE-*co*-DA), and PDA sheets using a droplet shape analysis system (DSA-25, KRÜSS, Hamburg, Germany) to evaluate the surface compatibility of polymer gels with PTFE. Contact angles were measured using water, diiodomethane, and hexadecane as dropping reagents. The surface tension components of each liquid are shown in Table 1. The test drop volume was 0.015 µL. At least five repeat measurements were performed for each sample. Based on the static contact angle values measured with the three test liquids, the surface free energy ($\sigma_{s}$) of the solid surfaces—PDEAE, P(DEAE-*co*-DA), PDA, and PTFE—was derived from Young’s equation (Equation (2)) [24,25], where $\gamma_{sl}$ is the interfacial tension between solid and liquid, $\sigma_{l}$ is the surface free energy of the liquid, and θ is the contact angle. Here, $\gamma_{sl}$ is calculated using the Owens–Wendt Equation (3). The total $\gamma_{sl}$ is composed of a dispersive component and a polar component for both the solid and the liquid. Specifically, $\sigma_{s}$ and $\sigma_{l}$ represent the surface free energies of the solid and the liquid, respectively; $\sigma_{s}^{d}$ and $\sigma_{l}^{d}$ denote their dispersive components, while $\sigma_{s}^{p}$ and $\sigma_{l}^{p}$ denote their polar components. To determine $\sigma_{s}$, three liquids with a known $\sigma_{l}^{d}$ and $\sigma_{l}^{p}$—distilled water, diiodomethane and hexadecane—were used to measure θ (Table 1) and apply the Owens–Wendt–Rable–Kaelble (OWRK) method (Equation (4)). Note that $\sigma_{s}=\sigma_{s}^{d}+\sigma_{s}^{p}$ and $\sigma_{l}=\sigma_{l}^{d}+\sigma_{l}^{p}$.

(2) $$\sigma_{s}=\gamma_{sl}+\sigma_{l}\cdot \cos\theta$$

(3) $$\gamma_{sl}=\sigma_{s}+\sigma_{l}-2\left(\sqrt{\sigma_{s}^{d}\cdot \sigma_{l}^{d}}+\sqrt{\sigma_{s}^{p}\cdot \sigma_{l}^{p}}\right)$$

(4) $$\left(1+\cos\theta \right)\sigma_{l}=2\sqrt{\sigma_{s}^{d}\cdot \sigma_{l}^{d}}+2\sqrt{\sigma_{s}^{p}\cdot \sigma_{l}^{p}}$$

The work of adhesion ($W_{a}$), an ideal quantity that excludes dissipative contributions, was calculated from the surface free energies according to the following Equation (5) [22,26,27,28].

(5) $$W_{a}=\sigma_{l}(1+\cos\theta )=2\sqrt{\sigma_{s}^{d}\cdot \sigma_{l}^{d}}+2\sqrt{\sigma_{s}^{p}\cdot \sigma_{l}^{p}}+2\sqrt{\sigma_{s}^{h}\cdot \sigma_{l}^{h}}$$

Here, $\sigma_{s}^{h}$ and $\sigma_{l}^{h}$ denote the contributions of the hydrogen-bonding components to the surface free energies of the solid and the liquid, respectively.

#### 2.3.5. Dynamic Mechanical Analysis (DMA)

1. Evaluation of viscoelastic properties

DMA was performed using a dynamic viscoelasticity analyzer (RSA-G2, TA Instruments, New Castle, DE, USA) to evaluate the viscoelastic properties of the three adhesive gels (PDEAE, P(DEAE-co-DA), and PDA). Samples were laser-cut into disks with a diameter of 15 mm and a thickness of 2 mm. Each disk was placed under a 15 mm diameter parallel plate with a gap of 0.8–2.0 mm. The measurements were conducted at 25 °C with a strain amplitude of 0.05% using frequency sweep tests over the range 1–100 Hz (corresponding to angular frequencies of 6.28–628.32 rad/s). The storage modulus (G′), loss modulus (G″), loss factor (tan *δ*), and complex viscosity ($\eta^{*}$) were measured for the PDEAE, P(DEAE-co-DA), and PDA gels.

2. Creating master curves and calculation of relaxation time (*τ*) and activation energy (*E_a_*)

To construct a master curve, an appropriate strain amplitude within the linear viscoelastic region was selected first. Strain sweep tests were then performed over a strain range of 0.001% to 10% at 25 °C and 1 Hz on the PDEAE, P(DEAE-co-DA), and PDA gels. From these results, a suitable strain amplitude within the linear viscoelastic region was chosen for each gel.

The master curves for each gel sample were constructed from frequency sweep tests based on the temperature–time conversion law. *τ* for the flow transition was estimated from the crossover points of G′ and G″ in the master curves at temperatures ranging from 25 °C to 165 °C. At the crossover frequency ($\omega_{c}$), the value of *τ* is given by ${\tau =1/\omega }_{c}$.

The $E_{a}$ was calculated using the Arrhenius equation (Equation (6)), which can also be expressed as Equation (7) [29,30]. Where $\tau^{*}\left(T\right)$ is relaxation time at temperature *T* (K) and $\tau_{0}^{*}$ is the relaxation time at the reference temperature; here, we use 25 °C (298 K); *R* is the universal gas constant (8.314 J/(mol∙K)). A plot of ln$\tau^{*}\left(T\right)$ against 1/T is known as an Arrhenius plot, $E_{a}$ was then derived from the slope of the linear regression fit to the data, where $E_{a}=slope\times R$.

(6) $$\tau^{*}\left(T\right)=\tau_{0}^{*}e^{{-E}_{a}/RT}$$

(7) $$\ln\tau^{*}\left(T\right)=\ln\tau_{0}^{*}-\frac{E_{a}}{RT}$$

#### 2.3.6. Differential Scanning Calorimetry (DSC)

The melting and crystallization behavior of the adhesive gels were studied on a DSC measuring machine (M-DSC Q-2000, TA Instruments, New Castle, DE, USA) operating under a nitrogen flow. Samples of about 5 mg were heated from 20 °C to 150 °C at a heating rate of 10 °C/min, held for 1 min, then cooled to −50 °C at a cooling rate of 5 °C/min and held for 1 min, and finally reheated again to 150 °C at a rate of 10 °C/min.

## 3. Results and Discussion

### 3.1. Adhesive Strength and Peel Energy of Gels to PTFE

**Figure 3:**
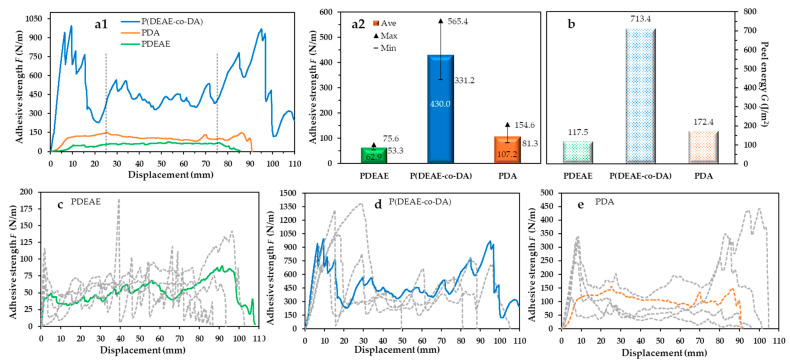
Adhesive strength *F* (peel force per unit width) versus displacement plots obtained from 90° peel tests for three gels adhesive to PTFE (**a1**,**c**–**e**). (**a1**) Comparison of typical force–displacement plots for PDEAE (green), P(DEAE-*co*-DA) (blue), and PDA (orange), (**a2**) maximum, mean and minimum adhesive strength values for each gel, calculated over the displacement range of 25 mm–75 mm. (**b**) Peel energy *G* for each gel, calculated from the total peel energy and peeled area using the force–displacement data for (**c**) PDEAE, (**d**) P(DEAE-*co*-DA), and (**e**) PDA (five specimens per gel, grey dashed lines represent the other four samples). The green, blue, and orange lines in (**c**–**e**) were selected as representative plots for PDEAE, P(DEAE-*co*-DA), and PDA, respectively, and are shown in (**a1**).

The adhesive strength *F* (peel force per unit width) versus displacement plots obtained from the 90° peel tests for the three gels that are adhesive to PTFE, and the peel energy *G* for each gel, calculated from the peel energy and peeled area using the force–displacement data, are shown in Figure 3. P(DEAE-*co*-DA) exhibited a much higher adhesive strength compared to PDEAE and PDA (Figure 3(a1)). Figure 3(a2) summarizes the average adhesive strengths. The average adhesive strength of P(DEAE-*co*-DA) was 430.0 N/m, which was significantly higher than 62.9 N/m for PDEAE and 107.2 N/m for PDA. The adhesive strength of P(DEAE-*co*-DA) was approximately 6.8 times that of PDEAE and 4 times that of PDA. Figure 3c–e displays multiple measurement results for each gel, confirming that P(DEAE-*co*-DA) consistently demonstrates high adhesive strength. Furthermore, these curves showed large fluctuations, suggesting a “stick–slip phenomenon” where adhesion and slippage were repeated. Figure 3b shows the peel energy *G* required to fracture the adhesive interface. P(DEAE-*co*-DA) again recorded the highest value at 713.4 J/m^2^. This value was exceptionally high compared to 117.5 J/m^2^ for PDEAE and 172.4 J/m^2^ for PDA. This indicates that a significantly larger amount of energy is required for debonding, suggesting the formation of a more robust adhesive bond for the P(DEAE-*co*-DA) copolymer gel compared to the PDEAE and PDA homopolymer gels.

### 3.2. Mechanical Properties of Adhesive Gels

**Figure 4:**
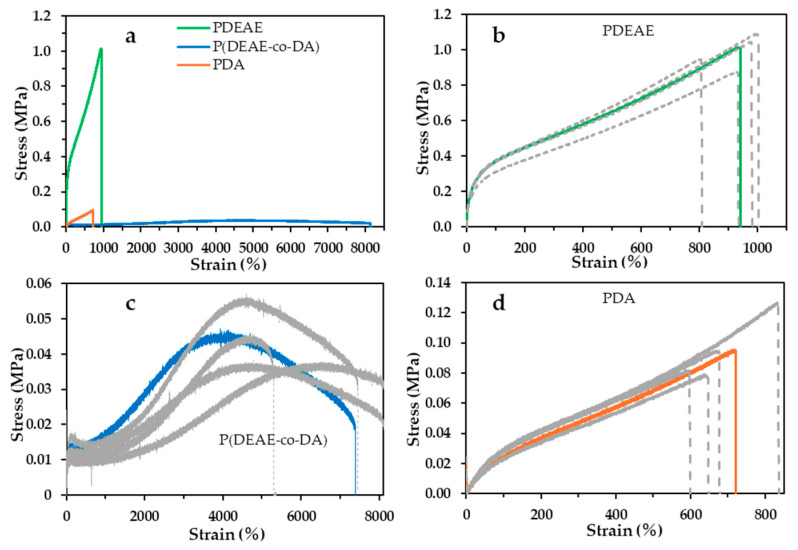
Tensile stress–strain curves for PDEAE, P(DEAE-*co*-DA), and PDA. (**a**) Comparison of typical curves for PDEAE, P(DEAE-*co*-DA), and PDA; curves for (**b**) PDEAE, (**c**) P(DEAE-*co*-DA), and (**d**) PDA obtained from five specimens. The green line in (**b**), blue line in (**c**), and orange line in (**d**) were selected for comparison in (**a**) as a typical curve for PDEAE, P(DEAE-*co*-DA), and PDA, respectively. The grey lines in (**b**–**d**) show the remaining 4 specimens.

Tensile tests were performed on the three polymer gels (PDEAE, P(DEAE-*co*-DA), and PDA). The tensile stress–strain curves of PDEAE, P(DEAE-*co*-DA), and PDA are presented in Figure 4. In Figure 4a, the ultimate tensile strength, breaking strain, toughness, and Young’s modulus of the three polymer gels are compared, and the average values of the mechanical properties are summarized in Table 2.

Among the three gels, PDEAE exhibited the highest ultimate tensile strength (0.993 MPa) and a relatively high Young’s modulus (2.171 MPa) (Figure 4b, Table 2), indicating excellent stiffness and load-bearing capacity. Additionally, its toughness (5.228 MJ/m^3^) was high, suggesting efficient energy absorption through a combination of high initial stiffness and substantial breaking strain, which prevents localized stress concentration. Although PDEAE achieved a breaking strain of 936%, its extensibility was lower than that of P(DEAE-*co*-DA). In contrast, P(DEAE-*co*-DA) displayed a significantly lower ultimate tensile strength (0.045 MPa), indicating poor resistance to tensile stress (Figure 4c, Table 2). However, its breaking strain was exceptionally high (7309%, 7.8 times that of PDEAE), demonstrating remarkable flexibility and extensibility. Its Young’s modulus was relatively low (1.609 MPa), suggesting easy deformation under stress. Although its toughness (2.185 MJ/m^3^) was moderate—lower than that of PDEAE—its high flexibility enables substantial energy absorption. PDA exhibited an ultimate tensile strength of 0.096 MPa, a breaking strain of 699%, and a Young’s modulus of 2.371 MPa (Figure 4d, Table 2), indicating greater stiffness than P(DEAE-*co*-DA) while maintaining moderate extensibility. However, its toughness (0.384 MJ/m^3^) was the lowest, reflecting inferior energy absorption capacity.

Overall, PDEAE excels in strength and toughness, P(DEAE-*co*-DA) is distinguished by its high flexibility and extensibility, and PDA offers a balance between stiffness and extensibility. When used as an adhesive tape, PDEAE would require the largest force to fracture, indicating the greatest resistance to breakage, while P(DEAE-*co*-DA) would show the largest amount of stretch at break.

### 3.3. Surface Structure of Three Adhesive Gels and PTFE

**Figure 5:**
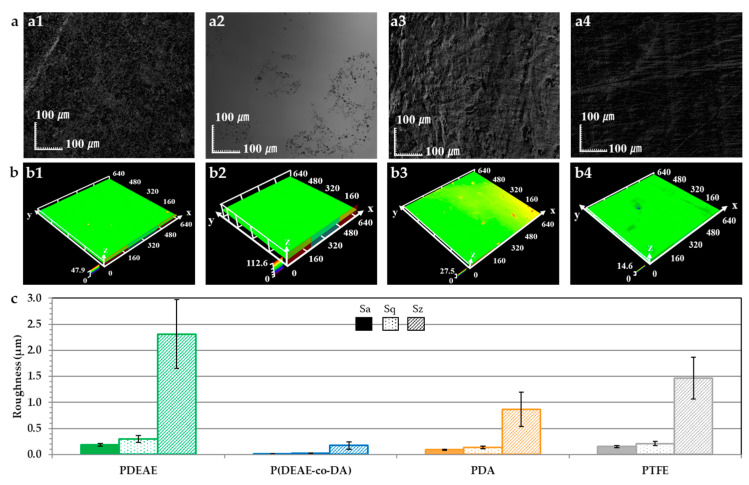
Surface characterization of three types of gels and PTFE. (**a**) Scanning-mode digital microscopy images and (**b**) 3D profilometry reconstructions for (**a1**,**b1**) PDEAE, (**a2**,**b2**) P(DEAE-*co*-DA), (**a3**,**b3**) PDA, and (**a4**,**b4**) PTFE. (**c**) Summary of 3D height parameters (Sa, Sq, Sz) quantifying surface roughness for PDEAE (green), P(DEAE-*co*-DA) (blue), PDA (orange), and PTFE (grey). Each bar includes error bars representing the standard deviation (±SD). (Note: Sa (arithmetic mean height) is defined as the arithmetic mean of the absolute deviations of the surface heights from the mean plane within the sampling area. Sq (root mean square height) represents the root mean square of these height deviations over the evaluated area. Sz (maximum height) is the vertical distance between the highest peak and the deepest valley within the defined measurement area. Sz quantifies the overall height variation of a surface and is mathematically equivalent to the sum of the maximum peak height and the maximum valley depth.).

The scanning-mode digital microscopy images and 3D profilometry reconstructions for the PDEAE, P(DEAE-*co*-DA), PDA gel sheets, and PTFE plate, obtained using a 3D measuring laser microscope, are shown in Figure 5a,b. The 3D height parameters—Sa, Sq, and Sz—used to evaluate the surface roughness are summarized in Figure 5c. Sa, Sq, and Sz indicate the deviations in surface height relative to the mean plane. Higher values of these parameters indicate greater surface roughness or unevenness.

As can be seen from these results, the PTFE plate used as the substrate exhibited a surface roughness with an Sa of 0.152 ± 0.021 µm, Sq of 0.212 ± 0.039 µm, and Sz of 1.465 ± 0.400 µm. Among the three synthesized gels, PDEAE showed the highest roughness values (Sa = 0.189 ± 0.024 µm, Sq = 0.301 ± 0.065 µm, Sz = 2.314 ± 0.659 µm), indicating a significantly rougher surface compared to the other samples. PDA exhibited intermediate roughness, with an Sa = 0.094 ± 0.009 µm, Sq = 0.133 ± 0.026 µm, and Sz = 0.871 ± 0.330 µm. In contrast, the only copolymer gel P(DEAE-*co*-DA) exhibited the smoothest surface, with an Sa of 0.018 ± 0.004 µm, Sq of 0.028 ± 0.007 µm, and Sz of 0.176 ± 0.071 µm, indicating a significantly smoother surface compared to the PDEAE, PDA and PTFE samples.

The high Sz value observed in PDEAE (2.314 ± 0.659 µm) suggests the presence of pronounced surface peaks or valleys. The copolymerization of DEAE with DA to form P(DEAE-*co*-DA) significantly reduced surface roughness compared to either PDEAE or PDA alone. The P(DEAE-*co*-DA) with the smoothest surface (Sz = 0.176 ± 0.071 µm) showed the highest *F* and *G*, suggesting that surface roughness did not contribute significantly to the interfacial adhesion between the gel and the PTFE.

### 3.4. Surface and Interfacial Properties of Adhesive Gels and PTFE

The measured contact angle (θ) values and the calculated surface free energy ($\sigma_{s}$) and work of adhesion ($W_{a}$) of PDEAE, P(DEAE-*co*-DA), PDA, and PTFE are shown in Table 3.

The θ on the surface of the polymer gels followed the trend PDA > P(DEAE-*co*-DA) > PDEAE, and for the probe liquids it followed the trend water > diiodomethane > hexadecane, indicating an increase of θ with probe-liquid polarity. These results imply that DA-derived moieties are strongly hydrophobic due to their long alkyl chains, thereby improving their adhesion to hydrophobic surfaces [31], while the DEAE-derived moieties are hydrophilic because of the presence of amino groups, which likely enhances specific interactions with the PTFE surface.

The $\sigma_{s}$ is an intrinsic property of a material’s surface, representing the free energy required to create a unit area of new surface. For the polymer gels, $\sigma_{s}$ increased in the order PDEAE > P(DEAE-*co*-DA) > PDA, and P(DEAE-*co*-DA) exhibited the largest dispersive contribution ($\sigma_{s}^{d}$ = 23.07 mJ/m^2^). The polar contribution ($\sigma_{s}^{p}$) followed the same trend (PDEAE > P(DEAE-*co*-DA) > PDA), reflecting the effect of the polar -CH_2_CH_2_-N(CH_2_CH_3_)_2_ groups derived from the DEAE monomer and indicating a dominant polar term. By contrast, PDA, which contains nonpolar long alkyl chains (-C_12_H_25_) derived from the DA monomer, showed the lowest $\sigma_{s}^{p}$ (0.13 mJ/m^2^). Compared with PTFE, PDEAE and P(DEAE-*co*-DA) exhibited higher $\sigma_{s}$ values, whereas PDA showed a lower $\sigma_{s}$. In general, higher $\sigma_{s}$ correlates with a greater affinity for adhesives and easier adhesion; accordingly, PDEAE and P(DEAE-*co*-DA) are expected to adhere more readily than PTFE and PDA.

**Figure 6:**
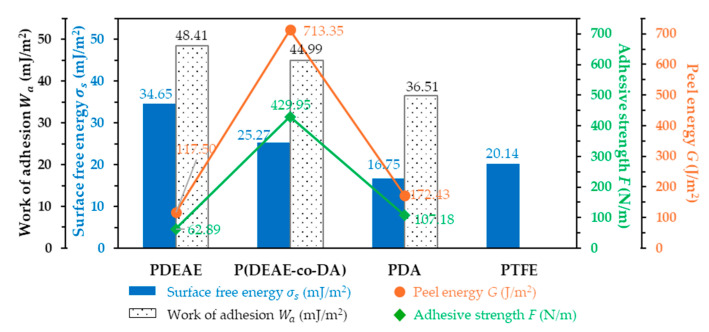
Comparison of calculated surface free energy $\sigma_{s}$ and work of adhesion $W_{a}$ with experimentally measured adhesive strength *F* and peel energy *G* for the gels and PTFE.

In interfacial chemistry, another energy quantity is the work of adhesion $W_{a}$. $W_{a}$ is the theoretical energy required to completely separate an interface between two materials and create new surfaces; it therefore describes the thermodynamic limit of adhesion under ideal (non-dissipative) conditions. We calculated the $W_{a}$ for each adhesive gel–PTFE interface. The $W_{a}$ values of PDEAE, P(DEAE-*co*-DA), and PDA on PTFE were 48.41, 44.99, and 36.51 mJ/m^2^, respectively, following the order PDEAE > P(DEAE-*co*-DA) > PDA (Table 3). Thus, under ideal thermodynamic conditions, the adhesive strength to PTFE is predicted to be PDEAE > P(DEAE-*co*-DA) > PDA, with PDEAE having the largest ideal adhesive strength. Notably, the calculated $W_{a}$ values were much smaller than the actual peel energy *G* measured in peel tests (Figure 3 and Figure 6). Moreover, the copolymer P(DEAE-*co*-DA) gel, which had the intermediate $W_{a}$, exhibited the highest measured adhesive strength *F* and peel energy *G*, while PDA (lowest $W_{a}$) showed a lower *F* and *G*, and PDEAE (highest $W_{a}$) unexpectedly showed the lowest *F* and *G*. This discrepancy arises because $W_{a}$ reflects only the interfacial intermolecular interactions (the ideal adhesive strength), whereas *G* is an experimental measure of actual adhesive performance that includes interfacial chemistry plus viscoelastic deformation and energy dissipation (fracture toughness) of the adhesive and adherends. Therefore, these results indicate the gels’ viscoelastic deformation and energy-dissipative mechanisms (e.g., physical entanglement, network defects) dominate the measured adhesive strength (*F* and *G*).

### 3.5. Viscoelastic Properties of Adhesive Gels

As mentioned above, the gels’ viscoelastic deformation and internal energy-dissipative mechanisms dominate the measured adhesive strength. Also, other researchers found that the viscoelasticity of polymer materials plays an important role in their adhesive properties [32,33,34,35]. The DMA was used to measure the G′, G″, tan *δ* and η* of PDEAE, P(DEAE-*co*-DA) and PDA in order to elucidate how each gel’s viscoelastic deformation and internal energy dissipation contribute to its adhesive strength.

**Figure 7:**
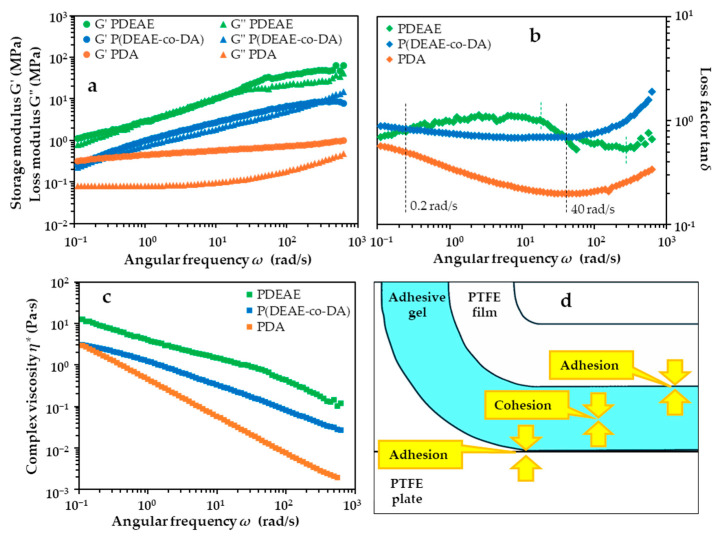
(**a**) Storage modulus G′ and loss modulus G″, (**b**) loss factor tan *δ*, and (**c**) complex viscosity η* of PDEAE, P(DEAE-*co*-DA) and PDA measured at 25 °C and under a strain of 0.05%. (**d**) Both the gel-PTFE adhesion and the gel cohesion affect the adhesive strength.

PDEAE (green) showed the highest G′ and G″ across most of the frequency range, P(DEAE-*co*-DA) (blue) was intermediate, and PDA (orange) was the weakest (lowest G′ and G″) and all three gels showed a positive frequency dependence (G′ and G″ increased with ω) (Figure 7a). Also, from the frequency dependence of the tan *δ* of the three gels (Figure 7b), we found that at a low *ω* (<0.2 rad/s) and a high *ω* (>40 rad/s) the tan *δ* of P(DEAE-*co*-DA) was higher than that of PDEAE, whereas at an intermediate *ω* PDEAE exhibited a higher tan *δ* than P(DEAE-*co*-DA). PDA showed the lowest tan *δ* over the entire frequency range. These results indicate that PDEAE contains highly mobile molecular chains, making it a viscosity-dominated gel, and its increase in tan *δ* at an intermediate *ω* suggests the presence of major energy-dissipating mechanisms, such as partial disentanglement of molecular chains and some local side-chain motions. On the other hand, PDEAE’s highest G′ (Figure 7a) and η* (Figure 7c) among the three gels indicates the highest intermolecular attractive forces and thus the strongest cohesion and molecular entanglement. Cohesion and adhesion along with interfacial surface are the two main contributors to adhesive strength (Figure 7d). However, because the G′ and η* were too high, the adhesion at the PDEAE–adherend (PTFE) interface was reduced, and consequently the overall adhesive strength decreased. This explains why the calculated $\sigma_{s}$ and $W_{a}$ were the highest while the measured *F* and *G* were the lowest among the three gels (Figure 6). For another homopolymer gel, PDA, the lowest tan *δ* and lowest G′ suggest the presence of relatively stable physical crosslinks, possibly due to the elongation of long alkyl side chains, which makes the elastic component dominant; however, its shape-retention ability is poor. The lowest η* suggests that PDA has a low viscosity and flows easily. Consequently, PDA stretches readily with weak force at the PDA–adherend (PTFE) interface. Therefore, PDA displayed weak adhesive properties, both theoretically and experimentally. For the copolymer gel P(DEAE-*co*-DA), because highly mobile molecular chains from the DEAE-derived moieties coexist with stable physical crosslinks from the DA-derived moieties, it behaved differently from the homopolymer gels PDEAE and PDA. P(DEAE-*co*-DA)’s moderate G′, moderate η* and moderate tan *δ* at an intermediate *ω* contribute to both strong adhesion to PTFE and strong internal cohesion; therefore, neither adhesive nor cohesive failure is readily initialted. As a result, the copolymer gel P(DEAE-*co*-DA) showed higher calculated $\sigma_{s}$ and $W_{a}$ than the homopolymer gels PDEAE and PDA, and also exhibited the highest measured *F* and *G* among the three gels (Figure 6).

**Figure 8:**
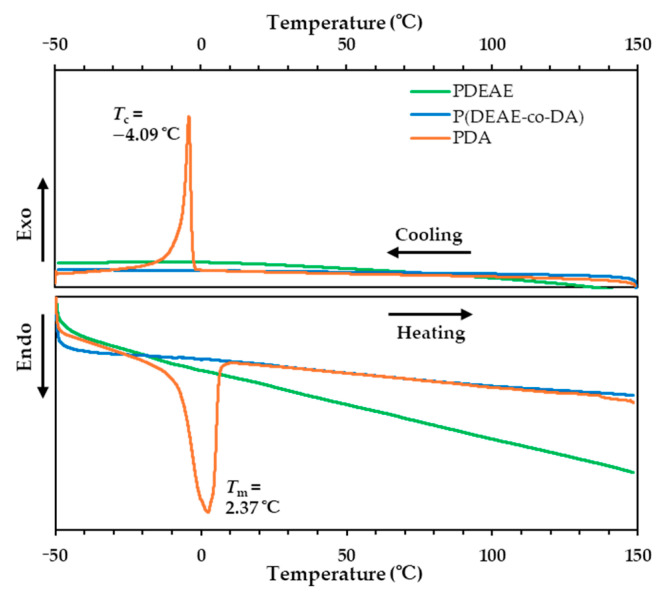
The DSC curves of PDEAE, P(DEAE-*co*-DA) and PDA recorded during cooling and the second heating cycle.

The DSC results (Figure 8) show that PDA had a clear melting peak (*T*_m_ = 2.37 °C) and a sharp crystallization peak (*T*_c_ = −4.09 °C), which can be attributed to partial crystallization of its long C12 alkyl side chains. Although *T*_m_ and *T*_c_ were below room temperature, these crystalline domains were likely to act as relatively stable physical crosslinks. P(DEAE-*co*-DA) did not display distinct melting or crystalliation peaks, similarly to PDEAE, despite containing DA-derived moieties. However, the baseline slope of P(DEAE-*co*-DA) matched that of PDA, whereas PDEAE showed a larger baseline slope. This indicates that PDEAE underwent significant endothermic heat flow associated with softenning of its highly mobile molecular chains. In contrast, the baseline slope of P(DEAE-*co*-DA) remained similar to PDA because the DA-derived relatively stable physical crosslinks and the DEAE-derived highly mobile molecular chains coexisted, balancing their thermal responses. In conclusion, the DSC results corroborate the interpretations given above regarding the differences in the calculated $\sigma_{s}$ and $W_{a}$ and the measured *F* and *G* for the three adhesive gels PDEAE, P(DEAE-*co*-DA) and PDA.

### 3.6. Relaxation Time and Activation Energy Evaluated from Master Curve

**Figure 9:**
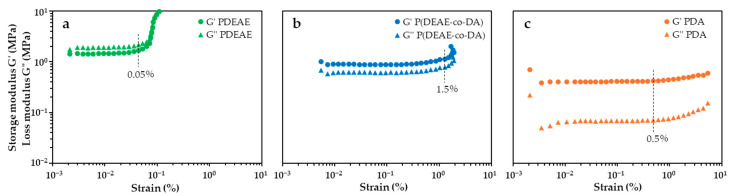
The strain dependence of storage modulus G′ and loss modulus G″ for (**a**) PDEAE, (**b**) P(DEAE-co-DA), and (**c**) PDA gels measured at 25 °C and 1 Hz (6.28 rad/s).

The strain dependence of the PDEAE, P(DEAE-co-DA), and PDA gels is shown in Figure 9. G′ and G″ tended to increase with increasing strain, and each gel exhibited a different linear viscoelasticity region. We selected strain amplitudes of 0.05% for PDEAE, 1.5% for P(DEAE-co-DA), and 0.5% for PDA to carry out DMA measurements and construct master curves for the three gels.

**Figure 10:**
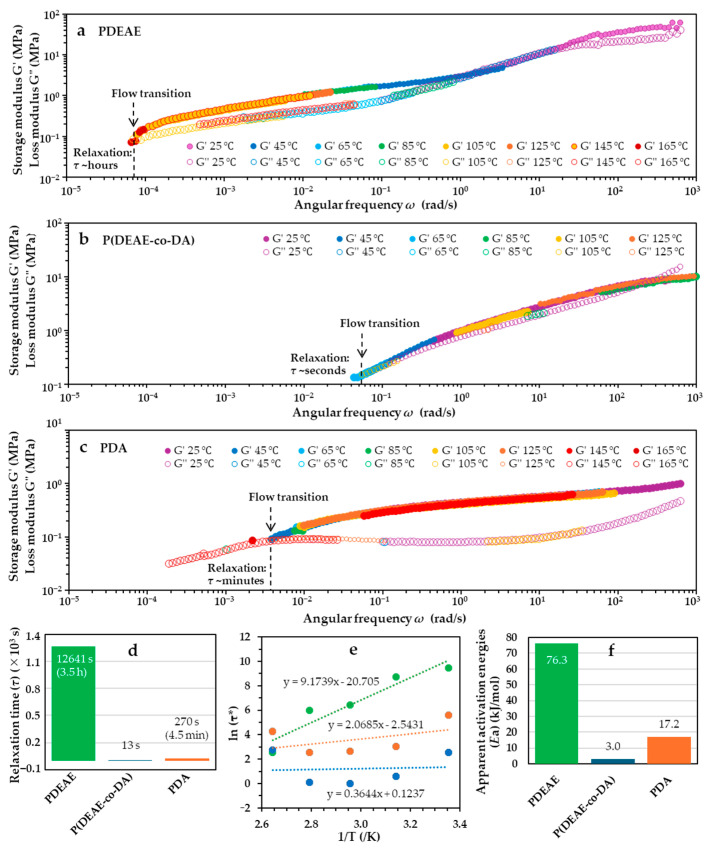
Master curves for (**a**) PDEAE, (**b**) P(DEAE-*co*-DA) and (**c**) PDA at a reference temperature of 25 °C, prepared by the time–temperature superposition treatment of storage modulus G′ and loss modulus G″ values obtained at 25, 45, 65, 85, 105, 125, 145, and 165 °C in frequency-dispersion tests [angular frequency ω = (6.28 × 10^–3^) to 628.32 rad/s] at an applied strain of 0.05% (PDEAE), 1.5% (P(DEAE-*co*-DA)) or 0.5% (PDA). (**d**) Relaxation times *τ* for the flow transition of PDEAE, P(DEAE-*co*-DA) and PDA, estimated from the crossover points of G′ and G″ at various temperatures (25 to 165 °C) (**a**–**c**). (**e**) Arrhenius plots of the intersection frequencies (*f* = ω/2π) for the G′ and G″ curves of PDEAE (green), P(DEAE-*co*-DA) (blue) and PDA (orange) (**a**–**c**). (**f**) Activation energies $E_{a}$ of PDEAE, P(DEAE-*co*-DA) and PDA, estimated from the Arrhenius plots (**e**).

On the basis of the frequency-dispersion properties of the G′ and G″ values at 25 °C to 165 °C with ω = (6.28 × 10^–3^) to 628.32 rad/s, we estimated the relaxation times *τ* for the flow transition of PDEAE, P(DEAE-*co*-DA), and PDA from their master curves at a reference temperature of 25 °C (Figure 10a–c), and the resulting *τ* values, plotted in Figure 10d, were 12,641 s for PDEAE, 13 s for P(DEAE-*co*-DA), and 270 s for PDA, indicating timescales of hours, seconds and minutes, respectively. The very large *τ* of PDEAE suggests it behaves almost like a glass or hard rubber under applied deformation: the movement of its entire molecular chains are slow, which easily leads to certain stress concentrations. The peel rate of 300 mm/min (5 mm/s) used in the peel tests was rapid compared with the large *τ*. So, even though PDEAE showed the highest calculated $\sigma_{s}$ and $W_{a}$, PDEAE’s relatively slow molecular response to rapid peeling likely caused its low peel force (adhesive strength *F*) during fast peeling. In contrast, P(DEAE-*co*-DA) had a short τ of 13 s, indicating fast movement of molecular chains at the adhesive surface. This behavior can be attributed to the combination of relatively stable DA-derived physical crosslinks and the short-term mobility of DEAE-derived chains. As a result, the material can effectively absorb and dissipate energy during peeling, which yielded the high adhesion properties of P(DEAE-*co*-DA). PDA had a medium relaxation time (*τ* = 270 s), so its molecular chains can relax to some extent and dissipate energy during peeling, but they did not reach sufficient fluidity (rapid chain movement), thus PDA’s adhesion properties remained only moderate.

**Figure 11:**
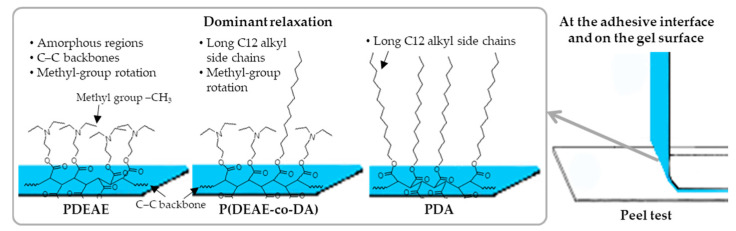
The dominant relaxation mechanisms for PDEAE, P(DEAE-*co*-DA) and PDA were inferred from their viscoelastic measurements and the estimated relaxation times *τ* and activation energies $E_{a}$.

Furthermore, the activation energies $E_{a}$ of PDEAE, P(DEAE-*co*-DA) and PDA were estimated from the Arrhenius plots (Figure 10e) and shown in Figure 10f. The $E_{a}$ is the energy required to drive the motion of the molecular chains. A high $E_{a}$ means the molecular chains move slowly and rearrangement is difficult, whereas a low $E_{a}$ indicates the molecular chains move relatively easily and rearrange quickly. The activation energies $E_{a}$ for PDEAE, P(DEAE-*co*-DA) and PDA were 76.3 kJ/mol, 3.0 kJ/mol, and 17.2 kJ/mol, respectively. From the literature, the $E_{a}$ for the thermal motion of an entire side chain is reported as 40~125 kJ/mol [36,37,38]; local torsional motion of the main backbone in the crystalline and amorphous regions is 40–85 kJ/mol [36]; the imine bond is 47.3 kJ /mol [39]; linear polyethylene is 26.1 kJ/mol [40,41], and the methyl-group rotational relaxation is less than 20 kJ/mol [37]. Based on these values, the dominant relaxation in PDEAE is likely derived from motions of the amorphous regions, entire chains including side chains and the main carbon–carbon (C–C) backbones like polyethylene, as well as methyl-group rotation (Figure 11). In PDA, relaxation was probably dominated by the motion of long C12 alkyl side chains, which need lower $E_{a}$ than the main C–C backbones. In P(DEAE-*co*-DA), the dominant relaxation likely involved the long C12 alkyl side chains and methyl-group rotation. These dominant relaxation mechanisms are consistent with and help explain the viscoelastic properties measured in Section 3.4: PDEAE is characterized by highly mobile molecular chains, PDA by relatively stable physical crosslinks, and P(DEAE-*co*-DA) by a combination of both behaviors.

The high $E_{a}$ of PDEAE means more energy is required to mobilize its molecular chains, which is consistent with its long *τ* and indicates slow chain mobility. Although PDEAE’s high G′ (Figure 9a) indicates strong cohesion, its high $E_{a}$ and long *τ*, that is, slow chain mobility and a large energy barrier to motion, reduce its ability to dissipate energy at the adhesive interface during peeling, thereby lowering adhesion. A good balance of high adhesion and high cohesion produces high adhesive strength (Figure 7d); PDEAE’s imbalance (high cohesion but poor dissipative response), as evidenced by its high $E_{a}$ and long *τ*, is therefore considered the main reason for its low measured *F* and *G*. The low $E_{a}$ of P(DEAE-*co*-DA) enabled good mobility and rapid motion of its molecular chains, which produces flexible adhesion; this is also consistent with its short *τ*. Moreover, its G′ (Figure 9a) was not low, indicating good cohesion. This led to the well-balanced adhesion and cohesion result in the high measured *F* and *G* of P(DEAE-*co*-DA) (Figure 6). PDA showed intermediate $E_{a}$ and *τ*, indicating a medium ability for chain mobility and hence some degree of adhesion. However, its G′ (Figure 9a) was the lowest among the three gels, indicating the lowest cohesion. The poor cohesion and intermediate adhesion caused PDA to exhibit only moderate adhesive properties in the peel tests.

## 4. Conclusions

We synthesized three polymer gels of PDEAE, P(DEAE-*co*-DA) and PDA, and found the copolymer gel P(DEAE-*co*-DA) had high pressure-sensitive adhesion to PTFE. P(DEAE-*co*-DA) recorded the highest adhesive strength (*F* = 430.0 N/m) and the highest peel energy (G = 713.4 J/m^2^) compared to the homopolymer gels PDEAE and PDA. The mechanical property measurements showed that PDEAE excels in strength and toughness, P(DEAE-co-DA) is distinguished by its high flexibility and extensibility, and PDA offers a balance between stiffness and extensibility. The surface structure measurements showed that P(DEAE-*co*-DA), which had the smoothest surface (Sz ≈ 0.176 µm), showed the highest *F* and *G*, suggesting that surface roughness did not contribute significantly to the interfacial adhesion between the gels and PTFE.

The surface free energy $\sigma_{s}$ and work of adhesion $W_{a}$ values of PDEAE, P(DEAE-co-DA), and PDA on PTFE followed the order PDEAE > P(DEAE-co-DA) > PDA; thus, under ideal thermodynamic conditions, the adhesive strength to PTFE is predicted as PDEAE > P(DEAE-co-DA) > PDA. However, the calculated $W_{a}$ values were much smaller than the actual *G* measured in the peel tests. Moreover, the copolymer gel P(DEAE-co-DA), which had the intermediate $W_{a}$, exhibited the highest measured *F* and *G*. These results indicate that the gels’ viscoelastic deformation and energy-dissipative mechanisms (e.g., physical entanglement, network defects) dominate the measured *F* and *G*. The results of the further evaluation of the viscoelastic properties, relaxation times *τ* and activation energies $E_{a}$ evidenced that achieving high adhesive strength requires a good balance of high adhesion and high cohesion. Short *τ* and low $E_{a}$ enable good mobility and rapid motion of molecular chains, producing flexible adhesion through effective energy absorption and dissipation during peeling. On the other hand, a high G′ provides strong intermolecular attractive forces and thus good cohesion. The imbalance in PDEAE (strong cohesion (high G′) but poor adhesion (long *τ* and high $E_{a}$)) and in PDA (poor cohesion and intermediate adhesion) is considered the main reason for their low measured *F* and *G*. The well-balanced adhesion and cohesion of P(DEAE-*co*-DA) resulted in its high measured *F* and *G*.

In this work, we succeeded in developing a P(DEAE-*co*-DA) gel with high pressure-sensitive adhesion to PTFE, and we found that if the polymer gel’s molecular motion can be well-controlled, it is promising that the adhesion of the P(DEAE-*co*-DA) gel can be further improved. In the next phase of this research, we plan to perform block copolymerization to obtain P(DEAE-*co*-DA) gels with well-controlled molecular structures, and to investigate the effect of block copolymerization and monomer ratio on adhesive strength. Materials with a greater adhesion to PTFE are expected to be developed, and the adhesion mechanisms can be clarified further.

## Figures and Tables

**Table 1:** Surface tension components for distilled water, diiodomethane and hexadecane.

Surface Tension	Water	Diiodomethane	Hexadecane
Total $\sigma_{l}$ (mN/m)	72.8	50.8	27.5
Polar $\sigma_{l}^{p}$ (mN/m)	51.0	0	0
Disperse $\sigma_{l}^{d}$ (mN/m)	21.8	50.8	27.5

**Table 2:** Mechanical properties of PDEAE, P(DEAE-co-DA) and PDA.

Sample	Ultimate Tensile Strength (MPa)	Breaking Strain (%)	Toughness (MJ/m^3^)	Young’s Modulus (MPa)
PDEAE	0.993	936.0	5.228	2.171
P(DEAE-*co*-DA)	0.045	7309.0	2.185	1.609
PDA	0.096	699.0	0.384	2.371

**Table 3:** Contact angles, surface free energies and work of adhesion for gels and PTFE.

Sample	Contact Angle θ (°)						Surface Free Energy (mJ/m^2^)			Work of Adhesion (mJ/m^2^)
	Water		Diiodomethane		Hexadecane		$\sigma_{s}^{d}$	$\sigma_{s}^{p}$	$\sigma_{s}$	$W_{a}$
PDEAE	72.88	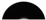	67.49	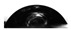	42.20	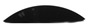	22.37	12.28	34.65	48.41
P(DEAE-*co*-DA)	91.52	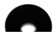	69.96	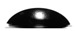	43.10	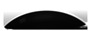	23.07	2.20	25.27	44.99
PDA	113.99	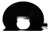	86.65	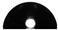	46.84	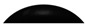	16.75	0.13	16.75	36.51
PTFE	109.98	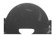	79.98	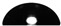	41.16	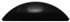	19.12	1.01	20.14	―

## Data Availability

The original contributions presented in this study are included in the article. Further inquiries can be directed to the corresponding authors.
